# Targeting IL-6 receptor mediated metabolic pathways to control Th17 cell differentiation and inflammatory responses

**DOI:** 10.3389/fimmu.2025.1568514

**Published:** 2025-08-27

**Authors:** Yazan Alwarawrah, Amanda G. Nichols, Isha Patel, Andréa B. Ball, Nancie J. MacIver

**Affiliations:** ^1^ Department of Pediatrics, Division of Pediatric Endocrinology and Diabetes, University of North Carolina, Chapel Hill, NC, United States; ^2^ Department of Nutrition, University of North Carolina, Chapel Hill, NC, United States; ^3^ Department of Molecular and Medical Pharmacology, University of California, Los Angeles, Los Angeles, CA, United States; ^4^ Department of Microbiology and Immunology, University of North Carolina, Chapel Hill, NC, United States

**Keywords:** interleukin-6 (IL-6), T cells, cellular metabolism, glycolysis, inflammation, immunometabolism, Th17

## Abstract

Interleukin-6 (IL-6) is a multifunctional cytokine that plays important roles in inflammation. Several studies have shown that IL-6 regulates various aspects of T cell function, including the differentiation of CD4^+^ T cells into the pro-inflammatory Th17 subset. Given the tight link between T cell metabolism and function, and the role of IL-6 in regulating cellular metabolism across tissues, we investigated the role of IL-6 signaling in Th17 cell metabolism. Using T cell specific IL-6 receptor (IL-6R) conditional knockout mice and littermate controls, we found that IL-6R signaling regulates the proportions of CD4^+^ and CD8^+^ T cells and drives CD4^+^ T cell differentiation into Th17 cells. We also found that IL-6R signaling is required for Th17 cell glycolytic metabolism. In T cell-specific IL-6R knockout mice, Th17 cells had reduced glucose uptake and glycolysis, as well as decreased expression of key glycolytic enzymes, while showing increased basal oxygen consumption. However, we also found that IL-6R signaling enhanced oxidative capacity and mitochondrial coupling efficiency in Th17 T cells. Importantly, inhibition of lactate dehydrogenase using FX11 selectively impaired Th17 cell differentiation with minimal effects on Treg cells. These findings suggest that targeting metabolic pathways regulated by IL-6R signaling can selectively inhibit inflammatory Th17 responses, offering a potential strategy for controlling IL-6 mediated inflammation.

## 1 Introduction

Interleukin-6 (IL-6) is a pleiotropic cytokine with roles in acute phase response, inflammation, and lymphocyte differentiation (1, 2). IL-6 is secreted by various cell types including macrophages, fibroblasts, adipocytes, and skeletal muscle cells during exercise (3, 4). At the molecular level, binding of IL-6 to either its transmembrane receptor (IL-6R) or its soluble receptor (sIL-6R) allows the cytokine-receptor complex to bind with gp130 on target cells, leading to activation of downstream Jak-STAT and Ras-ERK signaling pathways (5). Several cell types such as hepatocytes, monocytes, and T cells express the IL-6R, making them responsive to IL-6 signal (5). However, other cells that express gp130, such as smooth muscle cells and B cells, can respond to IL-6 signaling without IL-6R through sIL-6R (6, 7).

IL-6 has multiple effects on cell functions but is generally recognized as a pro-inflammatory cytokine that stimulates immune cell proliferation (8–10). Although IL-6 is essential for resolving bacterial and viral infections, increased levels of IL-6 have been associated with poor outcomes to certain infections such as SARS-CoV-2 (11, 12). Moreover, in autoimmune conditions, higher levels of IL-6 are associated with more severe disease outcomes (13, 14).

IL-6 signaling has been found to promote proliferation of T effector (Teff) cells in select contexts, increase differentiation of Teff cells into pro-inflammatory T helper 17 (Th17) cells, which produce the inflammatory cytokine IL-17, promote differentiation of T follicular helper cells, and inhibit the generation of T regulatory (Treg) cells (15, 16). T cell metabolism and function are tightly connected, and different metabolic programs are required for T cells to perform specific functions. In general, Teff cells primarily rely on glycolysis to meet their energy demands while naïve, Treg, and memory T cells predominantly utilize oxidative metabolism (17).

Although the effects of IL-6 on T cell function are well characterized, and IL-6 effects on the metabolism of metabolically active tissues and tumors have been investigated (18–20), the intrinsic effects of IL-6R signaling on T cell metabolism remain poorly understood. In this study, we investigated the effects of IL-6R signaling on T cell metabolism using a mouse model with T cell-specific knockout of the IL-6R (IL-6R cKO). We found that IL-6R induces glycolysis and enhances oxidative capacity in Th17 cells, and we identified several glycolytic enzymes that are upregulated by IL-6. Importantly, we also found that targeting lactate dehydrogenase selectively inhibited Th17 cell differentiation without affecting Treg cells. Taken together, these results highlight IL-6R as a key regulator of Th17 cell metabolism via metabolic mediators that can be selectively targeted for the treatment of IL-6-associated pathologies such as autoimmune disease and infection-associated inflammation.

## 2 Materials and methods

### 2.1 Animals

T cell-specific IL-6Rα conditional knockout (cKO) mice were generated by crossing CD4Cre transgenic mice (on a C57BL/6 background) with IL-6Rα-floxed C57BL/6 mice (Jackson Laboratory, Bar Harbor, ME). Mice were group housed (up to 5 per cage), maintained at ambient temperature, and given ad libitum access to food and water. Experimental mice were used at 8–12 weeks of age. All animal protocols were approved by the Institutional Animal Care and Use Committee at the University of North Carolina.

### 2.2 T cell isolation and differentiation

For the isolation of CD4^+^ T cells, spleens were disrupted into single cell suspensions in complete RPMI 1640 media (Corning, Corning, NY) using a 70 μm strainer. EasySep buffer (StemCell technologies, Cambridge, MA) was then added, and the cells were centrifuged at 1400 rpm for 5 min. Cells were then resuspended in EasySep buffer, and CD4^+^ T cells were isolated using the EasySep magnetic negative selection kit (StemCell technologies) following the manufacturer`s protocol.

CD4^+^ T cells were differentiated into Th1, Th17, and Treg cell subsets as previously described (21). Briefly, isolated CD4^+^ T cells were cultured for 3 days in 6 well plates [700,000 cells/well in 4 ml of complete RPMI (Th1 and Tregs) or IMDM (Th17)] precoated with 25 µg/ml anti-hamster antibodies (MP Biomedical, Solon, OH). The media contained a final concentration of 0.25 μg/ml anti-CD3 and 1 μg/ml anti-CD28 antibody (Biolegend). The following cytokines and antibodies were added to differentiate each subset; Th1: 40 ng/ml IL-12 (Biolegend), 100 IU/ml human IL-2 (Biolegend), 2 μg/ml anti–IL-4 (Biolegend); Th17: 10 ng/mL IL-6 (Biolegend), 0.3 ng/ml human TGF-β1 (Biolegend), 2 μg/ml anti–IL-4, 2 μg/mL anti-IFN-γ (Biolegend); and Treg: 10 ng/ml human TGF-β1, 100 IU/ml human IL-2, 2 μg/ml anti–IL-4, 2 μg/mL anti-IFN-γ.

### 2.3 Flow cytometry

Splenocytes were isolated from WT and IL-6R cKO mice. For the determination of the proportions of CD4, CD8, Th1, and Th17 cells, one million splenocytes were incubated in v bottom 96 well plate for 4.5 h in 200 µl complete media containing Brefeldin A (10ng/ml) (Biolegend), phorbol 12-myristate 13-acetate (PMA) (50 ng/ml) (Sigma-Aldrich, St. Louis, MO), and ionomycin (1 μg/ml) (Sigma-Aldrich). The cells were then washed with FACS buffer (2% FBS in PBS) and stained with BV605 Armenian Hamster anti-mouse CD3e (BD BioSciences, Franklin Lakes, NJ), Pacific Blue rat anti-mouse CD4 (Biolegend), and PE/cy7 rat anti-mouse CD8 (Biolegend) in FACS buffer containing rat anti-mouse CD16/CD32 (BD BioSciences). After 30 min incubation at 4°C, the cells were washed with FACS buffer, permeabilized, and fixed with Cytofix/Cytoperm kit (BD Biosciences) and stained with Alexa fluor 488 rat anti-mouse IFNγ, APC rat anti-mouse IL-17A following the manufacturer’s protocol.

For the determination of the proportions of Treg cells, one million splenocytes were incubated in a 96 well v-bottom plate in a volume of 100 μl of FACS buffer (2% FBS in PBS) containing rat anti-mouse CD16/CD32 (BD BioSciences), BV605 Armenian Hamster anti-mouse CD3e, pacific blue rat anti-mouse CD4, and Alexa fluor 488 rat anti-mouse CD25 (Biolegend) for 30 min at 4°C. The cells were then washed, fixed, and permeabilized using the Foxp3 Transcription Factor Staining Buffer kit (eBioscience) and stained for Foxp3 following the manufacturer’s protocol. All samples were analyzed on a BD FACSCanto II flow cytometer, and data were processed using FlowJo software (BD Biosciences).

For the determination of the proportions of differentiated Th1 and Th17 cells, 200,000 skewed cells were incubated in v bottom 96 well plate for 4.5 h in 200 µl complete media containing Brefeldin A (10ng/ml), PMA (50 ng/ml), and ionomycin (1 μg/ml). The cells were then washed with PBS and stained with Zombie green (Biolegend) for 10 min at room temperature, washed again, permeabilized and fixed with Cytofix/Cytoperm kit, and stained with PE rat anti-mouse CD4 (Biolegend), APC rat anti-mouse IFNγ, or IL-17A following the manufacturer’s protocol. For the determination of GLUT1 expression, anti-mouse GLUT1 (Abcam, Cambridge, United Kingdom) was added at the cytokine staining step. All samples were analyzed on a BD FACSCanto II or BD Accuri C6 flow cytometer.

For the determination of the proportions of differentiated Treg cells, 200,000 skewed cells were washed with PBS then stained with Zombie green (Biolegend) for 10 min at room temperature, washed again, fixed and permeabilized using the Foxp3 Transcription Factor Staining Buffer kit (eBioscience), and stained for PE rat anti-mouse CD4, PE/cy7 anti mouse CD25, and Foxp3 following the manufacturer’s protocol. All samples were analyzed on a BD Accuri C6 flow cytometer.

To assess STAT3 phosphorylation, 200,000 cells were incubated in Th17 differentiation media for 30 minutes. Cells were then collected and fixed with pre-warmed 1% PFA at 37°C for 10 minutes after which they were suspended in ice cold methanol and incubated at -20°C overnight. Cells were then washed with FACS buffer and stained with PE anti phospho-STAT3 and AF647 anti STAT3 antibodies (Biolegend) for 30 minutes at 4°C. After a final wash with FACS buffer, the cells were analyzed on a BD Accuri C6 flow cytometer.

### 2.4 Glucose uptake assay

Glucose uptake in T cells was assessed by tracking the accumulation of 2-Deoxy-2-[(7-nitro-2,1,3-benzoxadiazol-4-yl) amino]-D-glucose (2-NBDG) as we have previously described (22). In brief, cells were washed with glucose-free RPMI media containing 0.5% heat-inactivated FBS, then incubated for 30 min in the same media with 100 μM 2-NBDG (Thermo-Fisher) at 37°C. After that, the cells were washed with FACS buffer, stained for surface markers, and analyzed by flow cytometry.

### 2.5 Metabolic flux analysis

*In vitro* differentiated Th17 cells were washed with Seahorse XF RPMI 1640 media (Agilent, Santa Clara, CA) and plated at a density of 250,000 cells/well (50 μL) in a Seahorse XFe96 plate (Agilent) pre-coated with Cell-Tak following manufacturer instructions (Corning). The plate was then spun down at 200 rpm for 1 min, after which it was incubated for 30 min in a CO_2_ free humidified 37°C incubator. Following the incubation, 130 µL of Seahorse XF RPMI 1640 media was added, and the plate was incubated for an additional 20 min. For the glycolytic rate assay, proton efflux rate (PER) was measured using a Seahorse pro XFe96 metabolic flux Analyzer (Agilent). Data were collected under basal conditions and after the serial addition of the following drugs: 0.5 μM rotenone (Sigma-Aldrich) with 0.5 μM antimycin A (Sigma-Aldrich), followed by 20mM 2-deoxyglucose (Sigma-Aldrich). For the mitostress assay, oxygen consumption rate (OCR) was measured under basal conditions and after the addition of the following: 1μM oligomycin (Sigma-Aldrich), 0.5 μM flurorcarbonyl cynade phenylhydrazone (FCCP; Sigma-Aldrich), and 0.75 μM rotenone with 1.5 μM antimycin A.

### 2.6 Quantitative RT-QPCR of metabolic genes

RNA was isolated from *in vitro* differentiated Th17 cells using the RNeasy RNA extraction kit (Qiagen, Germantown, MD). From that, we synthesized cDNA using the iScript cDNA synthesis kit (BioRad, Hercules, CA). We retrieved the NCBI gene symbol for 230 metabolic genes from the KEGG signaling pathways database (23) spanning the glycolytic pathway, pentose phosphate pathway, TCA cycle, fatty acid synthesis, and oxidative phosphorylation. Using the NCBI gene symbol, we queried the PrimerBank database (24) for primers sets, selected the sets with experimental amplification evidence, and tested the sets for selectivity by running them against mouse transcript sequences using the Primer-BLAST (25). If we did not find primer sets with experimental evidence, new primers were designed using Primer-BLAST and tested using cDNA synthesized from universal mouse RNA (Agilent) (full list of primers is available in the **Supplementary Files**). We carried out the RT-qPCR reactions in 384 well PCR plates in a total volume of 4 µl containing 10 µM of reverse and forward primers, 2 µl of SensiFAST Sybr lo ROX RT-PCR mix (Bioline, Boston, MA), and 5 ng of template cDNA (1 µl). The reaction was run on a QuantStudio 5 RT-PCR thermo cycler (Thermo-Fisher).

### 2.7 Western blot

Differentiated Th17 cells from WT mice were washed with ice cold PBS and lysed with RIPA buffer (Sigma-Aldrich). Protein concentrations were measured using the Bio-Rad DC protein determination kit (Bio-Rad), and 50 μg of protein were resolved on a Bio-Rad TGX 8–16% gel (Bio-Rad) and transferred to PVDF membrane using TransBlot Turbo transfer system (Bio-Rad). Membranes were then blocked with EveryBlot blocking buffer (Bio-Rad) and probed with antibodies against the glycolytic enzymes using the cell signaling glycolysis antibody sampler kit (Cell Signaling Technologies, Danvers, MA), except for lactate dehydrogenase A where the antibody #2012 was used (Cell Signaling Technologies). We also probed for the mitochondrial OXPHOS complexes using the total OXPHOS rodent WB Antibody Cocktail kit (Abcam, Cambridge, UK). Rabbit anti-mouse β-actin (Cell Signaling Technology) was used as loading control. Images were acquired using ChemiDoc XRS+ imaging system (Bio-Rad). Band intensities were quantified using image Lab software (Bio-rad). Relative intensity was calculated by dividing band adjusted volume intensity by β-Actin band adjusted volume intensity.

### 2.8 Lactate assay

Media from T cell cultures (diluted 200X) and lactate standard (0-50 μg/ml) were diluted in lactate assay buffer (100 mM Tris HCl, 20 mM KCl pH 8.5), and 50 μl of the diluted sample was added to a 96 well black plate (Corning). After that, to each well, 50 μl of detection reagent (2.25 U/ml lactate dehydrogenase (Sigma-Aldrich), 2.7 U/ml Diaphorase (Innovative research, Novi, MI), 2 mM NAD (Sigma-Aldrich), and 28 μM Resazurin (Sigma-Aldrich) prepared in in assay buffer) was added. The plate was then incubated for 30 min at 37°C, after which fluorescence was measured at EX: 544/EM: 590 nm) using an Omega Fluostar plate reader (BMG LABTECH, Ortenberg, Germany).

### 2.9 *In vitro* inhibition of glycolysis

CD4^+^ T cells were isolated from WT mice and differentiated into Th17 or Treg cells for 72 hours in 96 well plates (20,000 cells per well in 200µl of media). After 48 hours from the start of the differentiation, the cells were treated with different concentrations of 2 deoxyglucose (2-DG; Cayman chemical, Ann Arbor, MI) or FX11 (Cayman chemical) for 24 hours. The Th17 cells were then collected and stained with zombie green fixable viability dye, PE CD4 (Biolegend) and APC rat anti-mouse IL-17A (eBioscience). Treg cells were stained with zombie green dye (Biolegend), PE CD4 (Biolegend), PE/Cy7 CD25 (Biolegend) and AF647 Rat anti-mouse Foxp3 (Biolegend), and viability and staining intensity were analyzed with flow cytometry.

### 2.10 Statistical analysis

Data were checked for normality using Shapiro–Wilk`s test. Unpaired t-tests with Welch’s correction were used if the data were normally distributed; if more than two groups were present, equal standard deviation was not assumed and the Brown-Forsythe and Welch ANOVA with Dunnett’s T3 multiple comparisons test was used. If samples failed the normality test, Mann Whitney nonparametric test was used; for multiple groups comparison, Kruskal–Wallis test was used with Dunn’s multiple comparisons test to compare group pairs. Any p value less than 0.05 was considered significant. T-test statistical analyses and grouped plot graphing were performed using GraphPad Prism 9 (GraphPad Software, Inc., La Jolla, CA).

## 3 Results

### 3.1 Loss of IL-6 receptor expression induces changes in T cell proportions and alters CD4^+^ T cell differentiation

**Figure 1:**
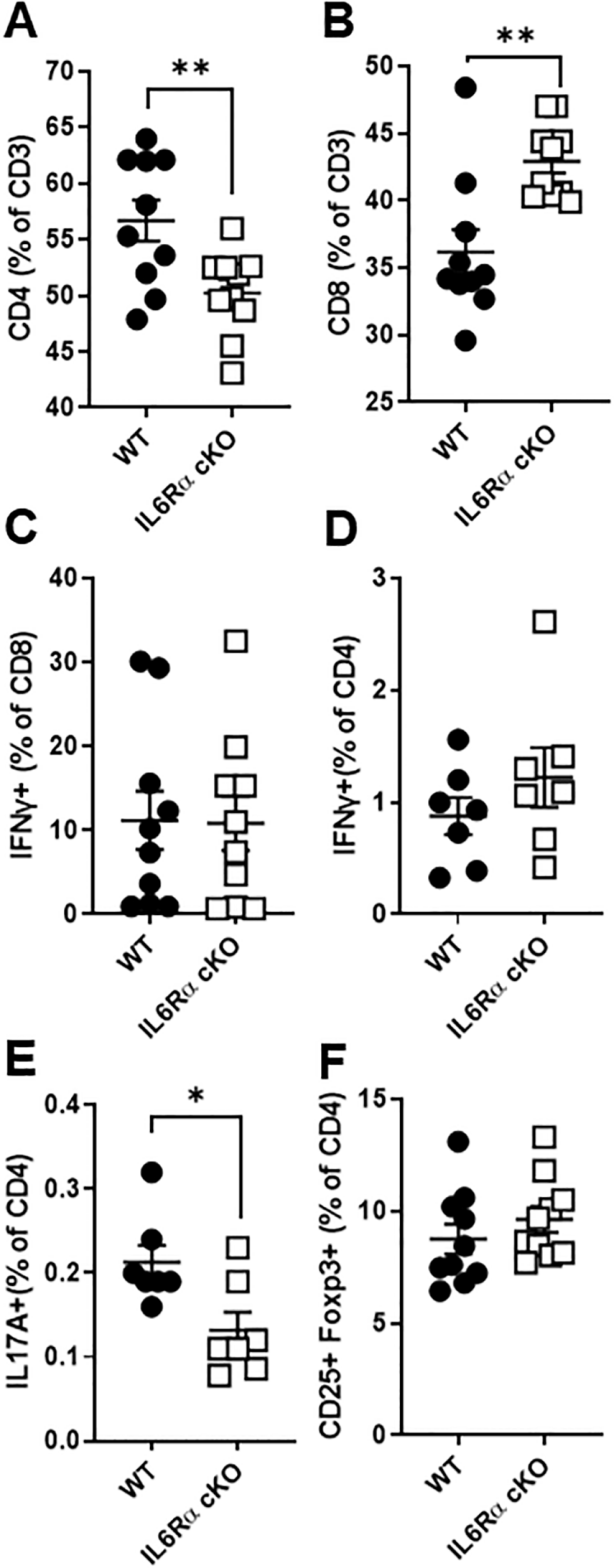
IL-6 receptor signaling influences T cell proportions and Th17 differentiation. Splenocytes were isolated from T cell-specific IL-6 receptor knockout mice and littermate controls. **(A, B)** Cells were stained for CD4 and CD8 expression to assess T cell proportions. **(C–F)** To evaluate effector cytokine production, cells were stimulated with ionomycin and PMA in the presence of Brefeldin A for 4.5 hours, followed by intracellular staining. Shown are the percentages of IFN-γ producing CD8^+^ T cells **(C)**, IFN-γ producing CD4^+^ T cells **(D)**, IL-17 producing CD4^+^ T cells **(E)**, and CD25^+^Foxp3^+^ regulatory CD4^+^ T cells **(F)**, as determined by flow cytometry. Data are pooled from 2–3 independent experiments. Error bars represent ± SEM. T-test with Welch’s correction or Mann Whitney test was used to compare groups depending on the normality of the distribution as judged by the Shapiro–Wilk test. n=7–10 mice/group; *p <0.05, **p <0.01.

**Figure 2:**
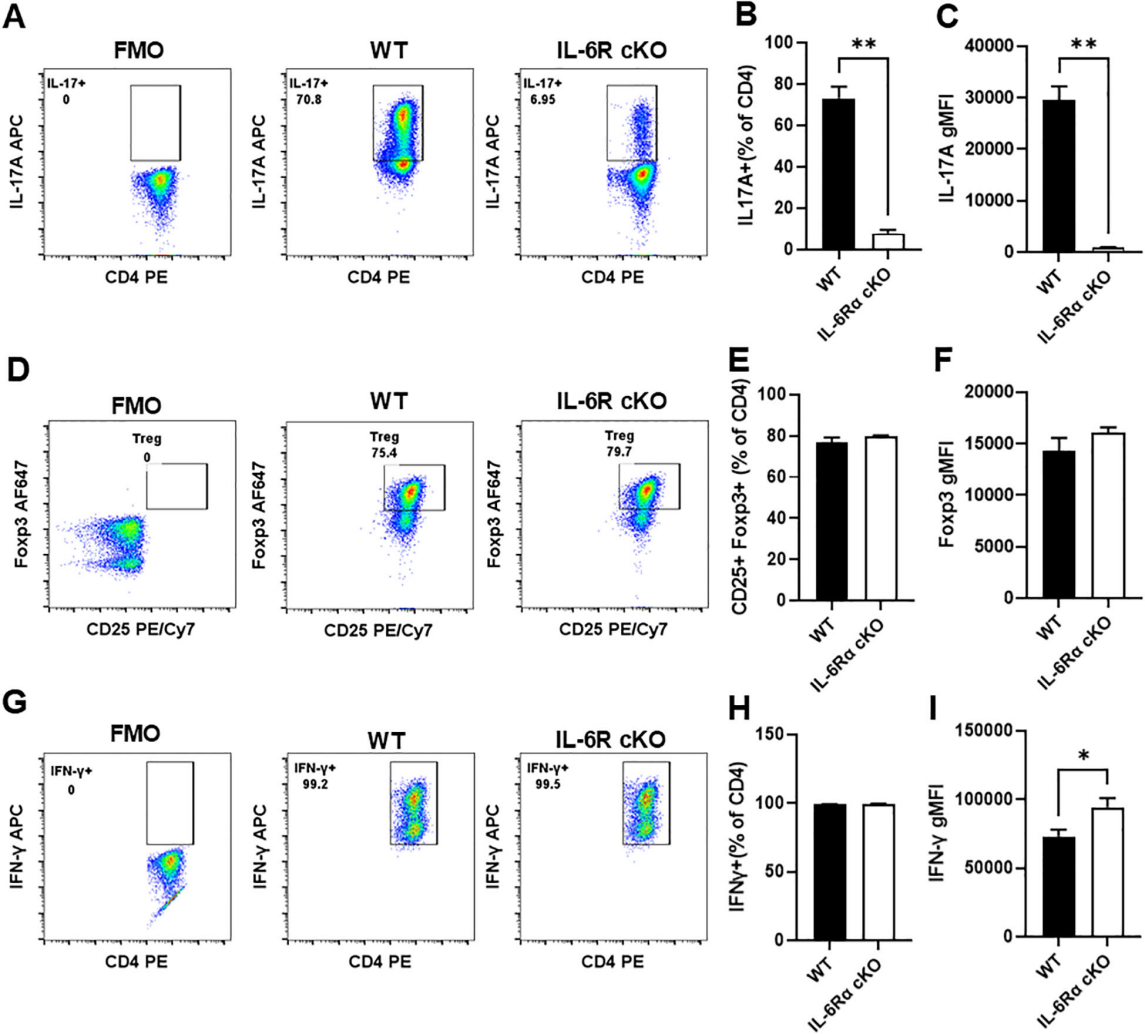
CD4^+^ cells from T cell-specific IL-6R KO mice fail to differentiate into Th17 cells *in vitro*. CD4^+^ T cells were isolated from T cell-specific IL-6 receptor knockout mice and littermate controls, differentiated *in vitro* toward Th17, Treg, or Th1 subsets, and analyzed by flow cytometry. **(A)** Representative plot for intracellular staining of IL-17 in cells differentiated toward Th17. **(B)** Proportion of differentiated Th17 cells producing IL-17. **(C)** Quantification of IL-17 expression in differentiated Th17 cells. **(D)** Representative plot for intracellular staining of CD25^+^Foxp3^+^ in cells differentiated toward Treg. **(E)** Proportion of differentiated Treg cells expressing CD25 and Foxp3. **(F)** Quantification of Foxp3 expression in differentiated Treg cells. **(G)** Representative plot for intracellular staining of IFN-γ in differentiated Th1 cells. **(H)** Proportion of differentiated Th1 cells expressing IFN-γ. **(I)** Quantification of IFN-γ expression in differentiated Th1 cells. Results are representative of 2–6 independent experiments, n=3 mice/group. Error bars represent ± SEM. T-test with Welch’s correction or Mann Whitney test was used to compare groups depending on the normality of the distribution as judged by the Shapiro–Wilk test. *p <0.05, **p <0.01.

To elucidate the role of IL-6R signaling in T cells, we generated T cell specific IL-6Rα conditional knockout (*CD4Cre^+^ IL-6Rα^fl/fl^*) and littermate controls (*CD4Cre^+^ IL-6Rα^wt/wt^*) by crossing IL-6R-flox mice with CD4-Cre mice. T cell specific IL-6Rα knockout mice (IL-6R cKO) showed a decrease in the proportion of splenic CD4^+^ T cells and an increase in CD8^+^ T cells (**Figures 1A, B**). We did not see any significant effect on the proportions of IFNγ^+^ CD8^+^ T cells (**Figure 1C**). Examination of CD4^+^ T cell subpopulations revealed a decrease in pro-inflammatory Th17 cells, while Th1 and Treg cell proportions remained unchanged (**Figures 1D–F**). We then examined the effect of losing IL-6R signal on CD4^+^ T cell differentiation *in vitro.* We found that CD4^+^ T cells from IL-6R cKO mice exhibited a profound defect in Th17 differentiation when activated by anti-CD3 and anti-CD28 antibodies in the presence of IL-6 and TGFβ1, as demonstrated by a striking decrease in IL-17 production compared to differentiated Th17 cells from littermate control mice (**Figures 2A–C**). These cells also showed reduced expression of RORγt (**Supplementary Figure 1A**) and increased expression of Foxp3 (**Supplementary Figure 1B**), suggesting a shift toward a Treg phenotype. In contrast, CD4^+^ T cells from IL-6R cKO mice showed no change in differentiation into Treg cells when activated by anti CD3 and anti CD28 antibodies in the presence of IL-2 and TGFβ1, as demonstrated by similar expression of Foxp3 and proportions of CD25^+^FOXP3^+^ T cells (**Figures 2D–F**). In differentiated Th1 cells from IL-6R cKO mice CD4^+^ T cells, we observed an increase in IFN-γ expression compared to Th1 cells differentiated from littermate control mice but without any significant change in the proportion of the differentiated Th1 cells (**Figures 2G–I**).

### 3.2 IL-6R induces glycolysis and enhances oxidative capacity in Th17 cells

**Figure 3:**
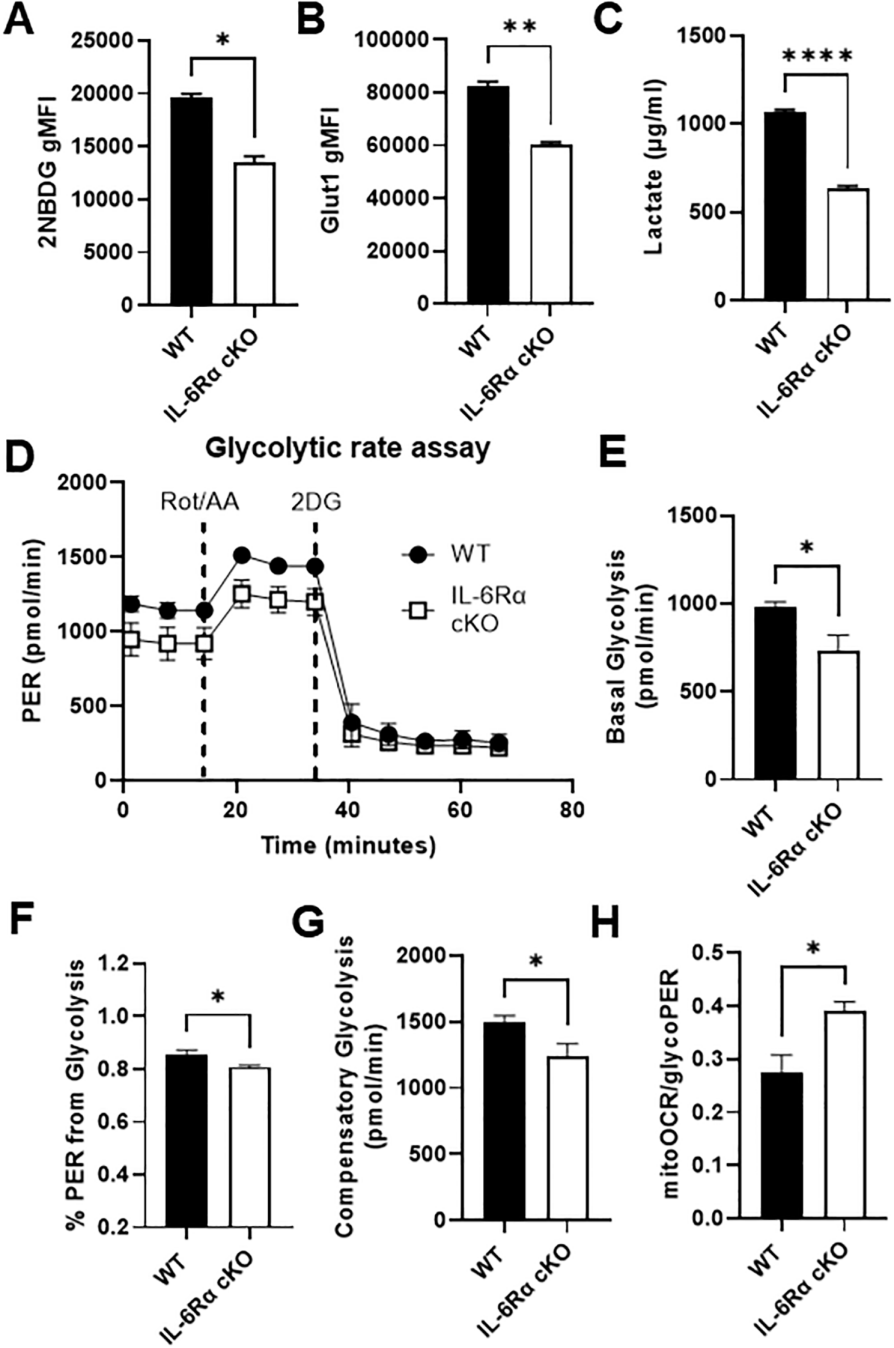
IL-6 receptor is required for Th17 cell glucose uptake and metabolism. CD4^+^ T cells were isolated from T cell-specific IL-6 receptor knockout mice and littermate controls and differentiated toward Th17 *in vitro*. **(A)** Cells were stained with 2-NBDG and analyzed flow cytometrically to determine glucose uptake. **(B)** Glut1 expression was measured by intracellular flow cytometry. **(C)** Lactate was measured in the culture media. **(D–H)** Glycolytic Rate Assay was performed using Seahorse extracellular flux analysis **(D)**, and the following parameters were calculated: glycolysis **(E)**, percent PER from glycolysis **(F)**, compensatory glycolysis **(G)**, and the ratio of mitoOCR to glycoPER **(H)**. Results representative of 2 independent experiments **(A–C)** or pooled from 2 independent experiments **(D–H)**, n=2–3 mice/group. Error bars represent ± SEM. T-test with Welch’s correction was used to compare groups depending on the normality of the distribution as judged by the Shapiro–Wilk test. *p < 0.05, **p < 0.01, ****p < 0.0001.

T cell metabolism and function are closely linked, such that different T cell subsets require and execute distinct metabolic programs to support their proliferation and function. Teff cells rely heavily on glycolysis to meet their energy demands, although glutamine metabolism is also important, while naïve, Treg, and memory T cells rely heavily on oxidative metabolism. Given that IL-6 is a strong inducer of Th17 differentiation, it would be reasonable to hypothesize that IL-6 induces metabolic changes in T cells that aid Th17 differentiation and function. Several reports have shown that IL-6 induces glycolysis in different types of cells including colorectal cancer cells, neuroblastoma cells, mouse fibroblasts and macrophages (18, 26–28); however, the role of IL-6 in driving T cell metabolism has not been investigated. To study the effect of IL-6 on T cell metabolism, we first compared glucose uptake between differentiated Th17 cells from IL-6R cKO mice and littermate controls by monitoring uptake of the fluorescent glucose analogue 2-NBDG using flow cytometry (**Figure 3A**). We also compared expression of the glucose transporter Glut1 (**Figure 3B**) and production of lactate (**Figure 3C**), between differentiated Th17 cells from control versus IL-6R cKO mice. In all of these parameters of glycolytic activity, we found a significant decrease in glucose uptake and utilization in Th17 cells differentiated from IL-6R cKO mice compared to Th17 cells from littermate controls. We then confirmed decreased glycolytic activity in Th17 cells differentiated from IL-6R cKO mice using a Seahorse Glycolytic Rate Assay (**Figure 3D**). Here, we observed a decrease in basal glycolysis (**Figure 3E**) and proton efflux rate (PER) from glycolysis (**Figure 3F**) in Th17 cells differentiated from IL-6R cKO mice compared to Th17 cells from littermate controls. Additionally, Th17 cells differentiated from IL-6R cKO mice had decreased compensatory glycolysis, which is the ability to induce glycolysis in response to blocking oxidative metabolism using rotenone and antimycin A compared to Th17 cells differentiated from littermate control mice (**Figure 3G**). Moreover, Th17 cells differentiated from IL-6R cKO mice had higher ratios of mitochondrial oxygen consumption rate (OCR) to glycolytic PER (**Figure 3H**). These results indicate that loss of IL-6R makes Th17 cells more oxidative than glycolytic.

**Figure 4:**
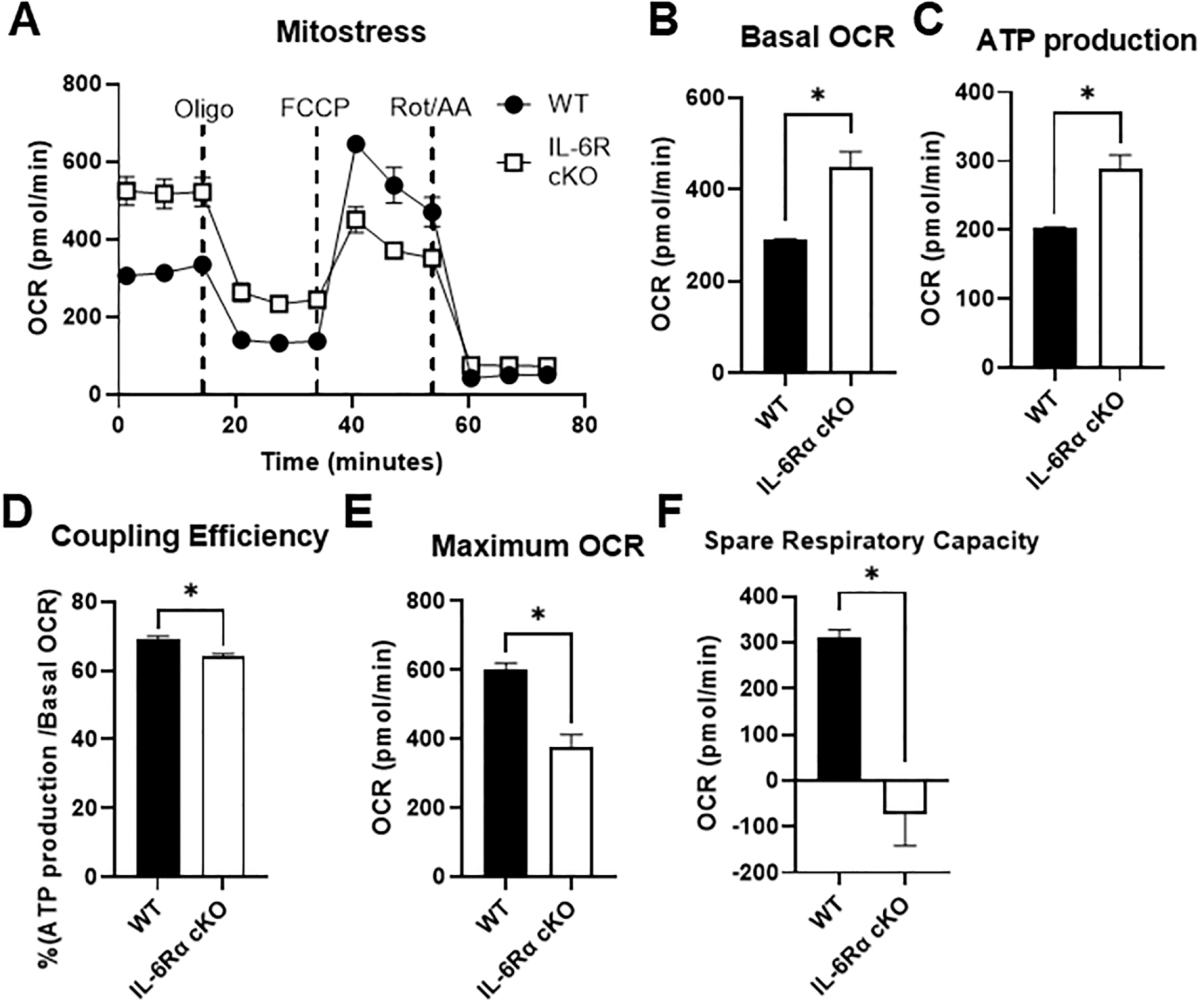
IL-6 receptor is required for Th17 cell oxidative capacity. CD4^+^ T cells were isolated from T cell-specific IL-6 receptor knockout mice and littermate controls and differentiated toward Th17 *in vitro*. **(A–F)** Mito Stress Test was performed using Seahorse extracellular flux analysis **(A)**, and the following parameters were calculated: basal OCR **(B)**, ATP production **(C)**, coupling efficiency **(D)**, maximum OCR **(E)**, and spare respiratory capacity **(F)**. Results representative of 2 independent experiments, n=3 mice/group. Error bars represent ± SEM. T-test with Welch’s correction was used to compare groups depending on the normality of the distribution as judged by the Shapiro–Wilk test. *p < 0.05.

To determine the effects of IL-6R deficiency on oxidative metabolism, we performed a Seahorse Mito Stress Test on differentiated Th17 cells from IL-6R cKO mice versus littermate controls (**Figure 4A**). Indeed, we found that basal OCR was significantly elevated in Th17 cells from IL-6R cKO mice compared to Th17 cells from littermate controls (**Figure 4B**), accompanied by an increase in mitochondrial ATP production (**Figure 4C**), but with lower efficacy as indicated by reduced coupling efficiency (**Figure 4D**). Interestingly, lack of IL-6R in Th17 cells led to a decrease in maximum OCR following the addition of oligomycin (an ATP synthase inhibitor) and FCCP (an electron transport chain uncoupler) (**Figure 4E**). This reduction resulted in diminished spare respiratory capacity (**Figure 4F**), indicating mitochondrial dysfunction and impaired oxidative capacity.

### 3.3 STAT 3 is a key mediator of IL-6 effects on T cell metabolism and IL-17 production

**Figure 5:**
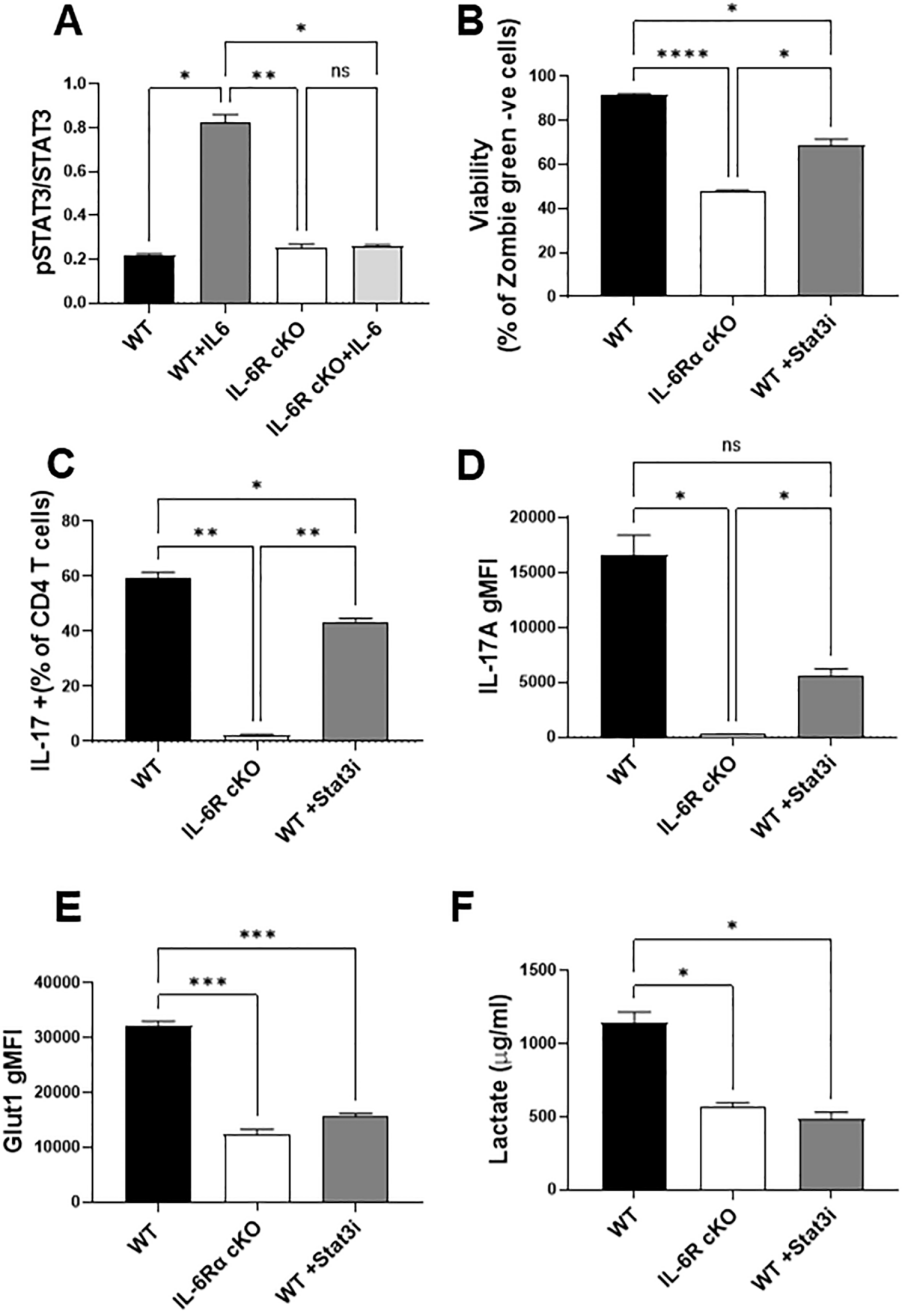
STAT 3 is a key mediator of IL-6 regulation of Th17 function and metabolism. **(A)** CD4^+^ T cells were isolated from T cell-specific IL-6 receptor knockout mice and littermate controls and differentiated toward Th17 *in vitro*. After 30 minutes, cells were fixed and stained for total STAT3 and phosphorylated STAT3. **(B–F)** CD4^+^ T cells were differentiated toward Th17 *in vitro* for 72 hours in the presence of absence of the STAT3 inhibitor Stattic V (Stat3i, 400 nM), and their viability **(B)**, Th17 differentiation **(C)** IL-17 production **(D)** and GLUT1 expression **(E)** were measured by flow cytometry. Media was collected and lactate was measured **(F)**. Results representative of 2 independent experiments n=3 mice/group. Error bars represent ± SEM. Brown-Forsythe and Welch ANOVA test were used to determine statistical significance and Dunnett’s T3 multiple comparisons test was used to compare groups depending on the normality of the distribution as judged by the Shapiro–Wilk test. *p < 0.05, **p < 0.01, ***p < 0.001, ****p < 0.0001.

IL-6 signals through multiple intracellular pathways, with STAT3 being a major downstream effector of its actions in T cells. To investigate the role of STAT3 activation, we assessed STAT3 phosphorylation in CD4^+^ T cells from wild-type (WT) and IL-6R cKO mice. We found that Th17 differentiation conditions containing IL-6 induced robust STAT3 phosphorylation in WT cells, but not in IL-6R cKO cells (**Figure 5A**), confirming that IL-6R is required for STAT3 activation in this context. To determine if the inhibition of STAT3 could recapitulate the effects of IL-6R deficiency on Th17 differentiation and metabolism, we treated WT CD4^+^ T cells with the STAT3 inhibitor Stattic V. We selected a concentration of the inhibitor that did not cause excessive cell death (**Figure 5B**). Inhibition of STAT3 partially but significantly reduced Th17 differentiation and IL-17 expression (**Figures 5C, D**), mimicking the phenotype observed in IL-6R cKO T cells. Consistent with this, STAT3 inhibition also decreased Glut1 expression to levels comparable to those seen in IL-6R-deficient Th17 cells (**Figure 5E**). Moreover, lactate production was similarly reduced upon STAT3 inhibition, again resembling the metabolic phenotype of IL-6R cKO T cells (**Figure 5F**). Together, these findings support a central role for STAT3 in mediating IL-6 dependent metabolic reprogramming and effector function in Th17 cells.

### 3.4 IL-6R induces changes in metabolic gene expression

**Figure 6:**
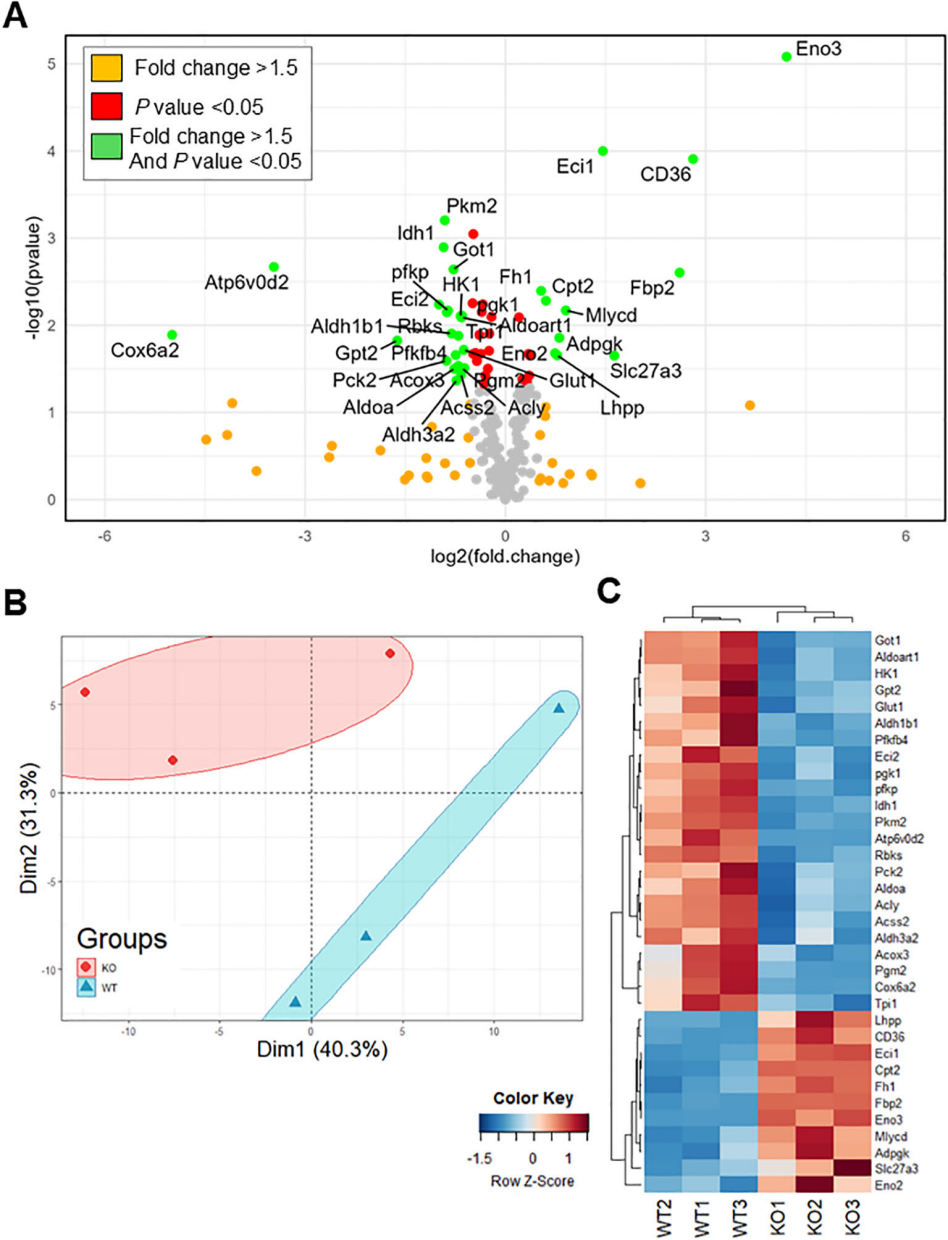
IL-6 receptor signaling induces broad changes in metabolic gene expression. CD4^+^ T cells were isolated from T cell-specific IL-6 receptor knockout mice and littermate controls and differentiated toward Th17 *in vitro*, after which RNA was isolated, and cDNA was synthesized. Samples were analyzed for the expression of 230 different metabolic genes spanning several metabolic pathways by RT-qPCR. **(A)** Volcano plot for all measured genes comparing expression in IL-6 receptor knockout mice to wildtype. **(B)** Principal component analysis for the expression of the metabolic genes comparing expression in IL-6 receptor knockout mice to wildtype. **(C)** Heat map of significantly changed genes with more than 1.5-fold difference (n=3 mice/group, z-score calculated based on 2^-ΔCt^).

IL-6R-induced changes in Th17 glycolysis and oxidative capacity suggest corresponding changes in the expression or activity of the enzymes that regulate these metabolic programs. To investigate this, we profiled the RNA expression of 230 metabolic genes in Th17 cells differentiated from IL-6R cKO mice and littermate controls using RT-qPCR (**Figure 6A**). The gene panel spanned key metabolic pathways including glycolysis, TCA cycle, pentose phosphate pathway, amino acid metabolism, fatty acid metabolism, and oxidative phosphorylation, as well as several glucose, amino acid, and fatty acid transporters. We observed significant differences in the metabolic gene expression profiles between Th17 cells from IL-6R cKO mice compared to Th17 cells from littermate controls (**Figure 6B**). Several genes were identified that were significantly upregulated or down regulated by IL-6R signaling (**Figure 6C**). For example, we found that IL-6R is required for the expression of several glycolytic genes (e.g., Slc2a1 (Glut1), Hk1, Pfkp and Pkm2), as well as genes encoding enzymes involved in amino acid metabolism (e.g., Got1 and Gpt2). At the same time, IL-6R signaling decreased the expression of genes involved in fatty acid transport (e.g., Slc27a3 and CD36). Additional genes were found to be highly induced by IL-6R that, to our knowledge, have not been previously implicated in IL-6R signaling in T cells, such as the vacuolar ATPase subunit Atp6v0d2 and the complex IV stabilizing subunit Cox6a2 (29).

**Figure 7:**
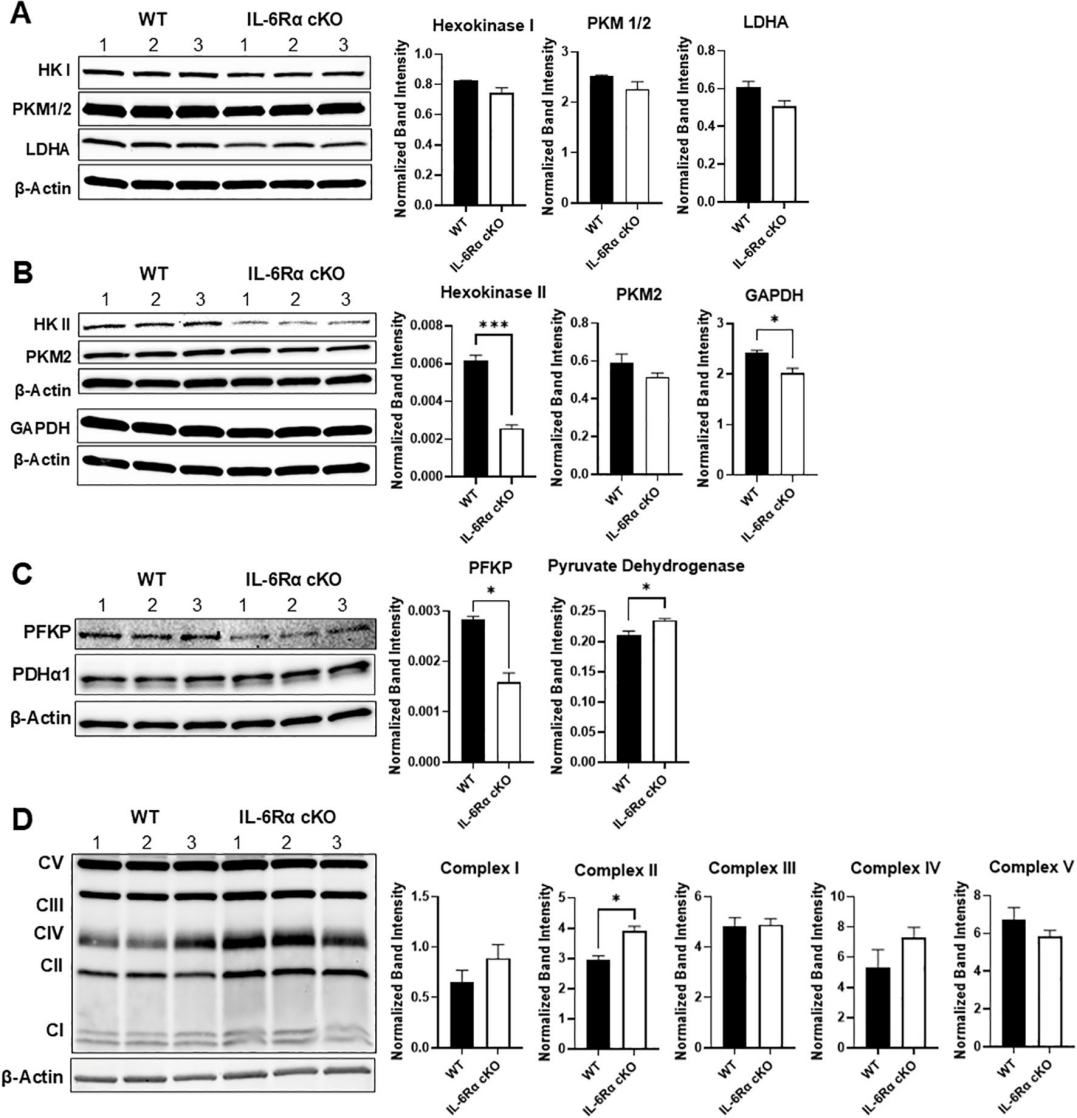
IL-6 receptor signaling induces the expression of glycolytic enzymes. CD4^+^ T cells were isolated from T cell-specific IL-6 receptor knockout mice and littermate controls and differentiated toward Th17 *in vitro*, after which cells were lysed and immunoblotted for several glycolytic proteins: hexokinase I, pyruvate kinase M1/2, and lactate dehydrogenase **(A)**, hexokinase II, pyruvate kinase M2, and glyceraldehyde phosphate dehydrogenase **(B)**, phosphofructokinase and pyruvate dehydrogenase **(C)** and expression of complexes in the mitochondrial electron transport chain, using the OXPHOS antibody cocktail **(D)**. Results representative of 2 independent experiments, n=3 mice/group. Expression calculated relative to actin. Error bars represent ± SEM. T-test with Welch’s correction or Mann Whitney test was used to compare groups depending on the normality of the distribution as judged by the Shapiro–Wilk test. *p < 0.05, ***p < 0.001.

At the protein level, we examined the expression of key glycolytic enzymes using immunoblotting. We found that the loss of IL-6R signaling led to significantly reduced protein levels of hexokinase II, glyceraldehyde-3-phosphate dehydrogenase, and phosphofructokinase, with a trend decrease in the expression of hexokinase I, pyruvate kinase and lactate dehydrogenase A (**Figures 7A–C**). We also examined the expression of complexes in the mitochondrial electron transport chain, using the OXPHOS antibody cocktail in Th17 cells differentiated from IL-6R cKO mice versus littermate controls (**Figure 7D**). Here, we found a significant increase in the expression of complex II in Th17 cells from IL-6R cKO mice compared to controls.

### 3.5 Blocking lactate dehydrogenase activity selectively inhibits Th17 differentiation

**Figure 8:**
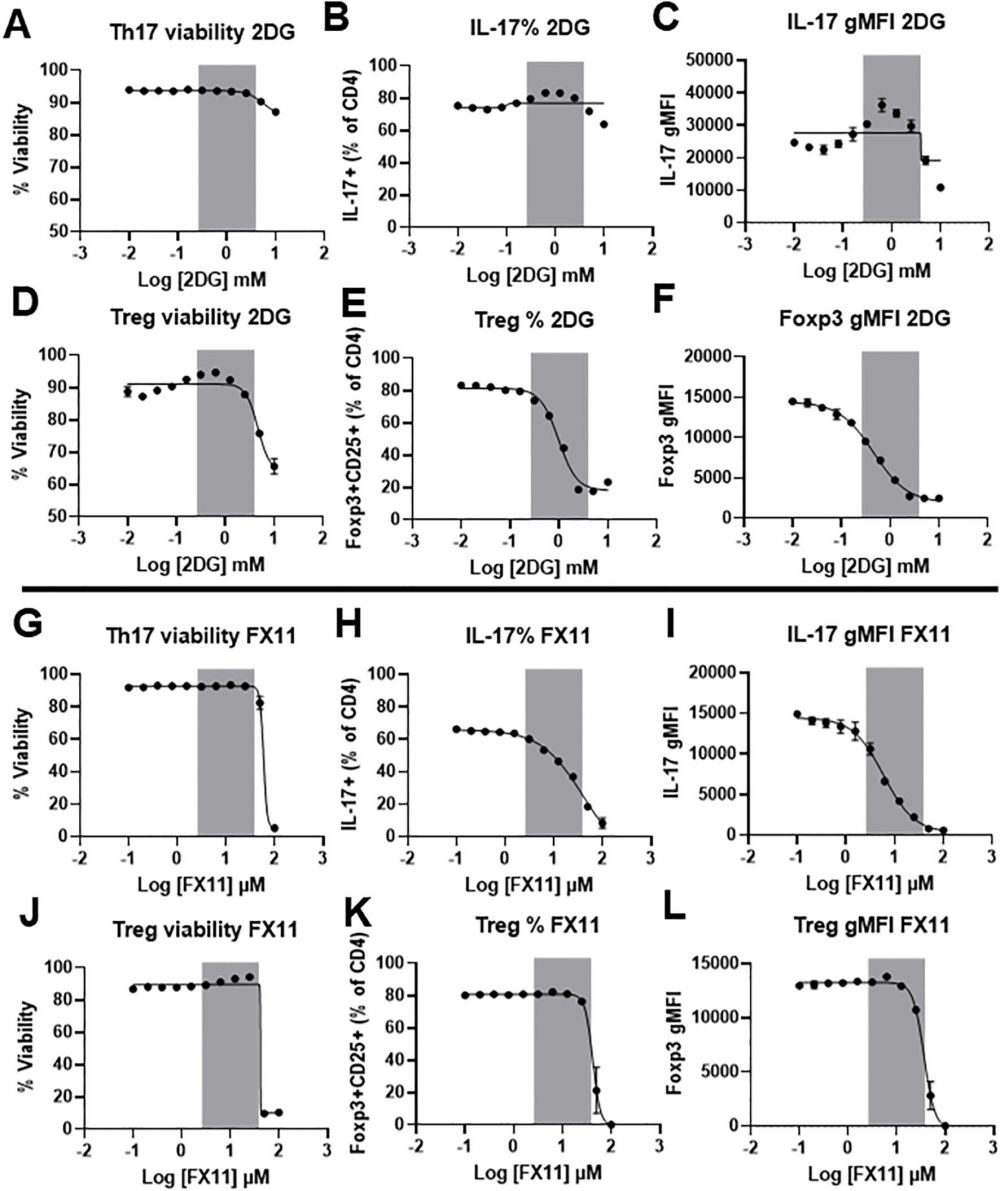
Blocking lactate dehydrogenase activity selectively inhibits Th17 differentiation. CD4^+^ T cells were isolated from wildtype C57BL/6 mice and differentiated *in vitro* toward Th17 or Treg subsets for 72 hours. Differentiated cells were then treated with increasing concentrations of 2DG or FX11 for an additional 24 hours. Cells were collected and stained for viability **(A, D, G, J)**, IL-17 expression **(B, C, H, I)**, or Foxp3 and CD25 expression **(E, F, K, L)**. Shaded area represents the concentration at which no significant effect on viability is noticed with changes in the differentiation of any of the two subsets. Results representative of two independent experiments, pooled from 3 mice/group. Sigmoidal 4PL non liner fit was used to create the regression line. Error bars represent ± SEM.

Our data thus far indicate that IL-6R signaling strongly induces the glycolytic pathway, with an increase in the expression of several glycolytic enzymes. We therefore hypothesized that glycolysis is critical for the induction of Th17 differentiation by IL-6, while less important for Treg cells, which depend more on oxidative metabolism and do not require IL-6R signal for differentiation. To test this hypothesis, we differentiated Th17 and Treg cells *in vitro* and inhibited glycolysis at two key points. First, we targeted the upstream step of glucose phosphorylation using 2-deoxyglucose (2-DG). Although 2-DG can be phosphorylated by hexokinase I and II into 2-deoxy-D-glucose-6-phosphate, it inhibits glucose-6-phosphate isomerase activity, preventing further metabolism and resulting in upstream blockade of glycolysis. We also targeted the downstream step of lactate dehydrogenase activity using the small molecule inhibitor FX11 (30). Differentiated Th17 and Treg cells from wildtype C57BL/6J mice were treated with either 2-DG or FX11 for 24 hours following differentiation. At high concentrations of 2-DG, Treg cell viability was more affected than Th17 cells (**Figures 8A, D**). At nontoxic concentrations of 2-DG (less than 500µM) we found that Treg differentiation (**Figure 8E**) and Foxp3 expression (**Figure 8F**) were significantly reduced compared to Th17 differentiation (**Figure 8B**) and IL-17 expression (**Figure 8C**). In contrast, when treating Th17 and Treg cells with nontoxic concentrations of FX11 (less than 25µM) (**Figures 8G, J**), Th17 differentiation (**Figure 8H**) and IL-17 expression (**Figure 8I**) were more reduced when compared to Treg differentiation (**Figure 8K**) and Foxp3 expression (**Figure 8L**) at the same FX11 concentration. These results suggest that inhibiting lactate dehydrogenase activity, a key downstream enzyme in the glycolytic pathway, can selectively inhibit Th17 differentiation, and may be used to strategically limit inflammation in the setting of disease.

## 4 Discussion

IL-6 is a pivotal regulator of immune responses, influencing various immune cell functions and playing a critical role in Th17 cell differentiation. Th17 cells are particularly important due to their role in autoimmune diseases and chronic inflammation. Given the broad impact of IL-6 on immune cell function, several inhibitors have been developed to target the IL-6R signaling pathway as a treatment for autoimmune diseases and to control viral infection-associated inflammation (31–33). However, due to the pluripotent function of IL-6 and the ability of any cell that expresses gp130 to conduct the IL-6 signal through the sIL-6R trans-signaling, targeting IL-6 directly can have widespread effects on the immune system. Better understanding of IL-6 signaling pathways downstream of IL-6R may lead to the development of more targeted therapies to modulate inflammation and autoimmunity.

While the role of IL-6 in regulating metabolism is well documented in various types of metabolic tissues, the effects of IL-6 on T cell metabolism have not been studied. Given that T cell function and differentiation are highly dependent on their metabolic phenotype, understanding the role of IL-6 in T cell metabolism is critical. In this study, we used a T cell specific IL-6R cKO mouse model to identify how IL-6R signaling affects T cell metabolism. Previous studies using IL-6R cKO mice found that loss of IL-6 signal impaired Th1 and Th17 immune responses (34) and reduced inflammation and diet-induced insulin resistance in the early stage of diet-induced obesity (35). Building on this work, we found that IL-6R signaling is essential for maintaining T cell homeostasis, as IL-6R cKO mice exhibited decreased frequencies of CD4^+^ and Th17 cells, increased CD8^+^ T cells, and no significant changes in Treg cell proportions. *In vitro*, IL-6R-deficient CD4^+^ T cells failed to efficiently differentiate into Th17 cells, as shown by their markedly reduced IL-17 production. This was accompanied by diminished RORγt expression and increased Foxp3 expression, suggesting a shift toward a Treg-like phenotype. The presence of residual IL-17–producing cells may reflect the activity of other cytokines such as IL-1, IL-21, and IL-23 (36), or the presence of soluble IL-6R in the culture media. In contrast, IL-6R-deficient CD4^+^ T cells retained normal Treg and Th1 differentiation capacities, although Th1 cells exhibited enhanced IFN-γ production.

Given the specific impairment in Th17 differentiation, we next examined how IL-6R signaling regulates Th17 cell metabolism. Utilizing metabolic flux analysis, we found that Th17 cells from IL-6R cKO mice were less glycolytic with lower glucose uptake, lower Glut1 expression, and decreased lactate production than Th17 cells from littermate controls. This was accompanied by an increase in basal oxidative metabolism but reduced oxidative capacity. These findings suggest that IL-6R signaling promotes both glycolytic flux and oxidative metabolic fitness in Th17 cells. In the absence of IL-6R signaling, Th17 cells may attempt to compensate by upregulating oxidative pathways, albeit ineffectively. Our data indicate that STAT3 is a key mediator of IL-6R-mediated metabolic programming. IL-6-induced STAT3 phosphorylation was abrogated in IL-6R-deficient T cells. Furthermore, pharmacologic inhibition of STAT3 phosphorylation partially recapitulated the metabolic and functional defects observed in IL-6R cKO cells, including reduced IL-17 production and diminished glycolysis. RNA and protein expression analyses further confirmed that IL-6R signaling induces the expression of several glycolytic enzymes, including Glut1, reinforcing the central role of STAT3 in this pathway.

To investigate if blocking glycolysis would mimic the effects of loss of IL-6R signal on Th17 cells, we used 2-DG and FX11 to block upstream and downstream portions of the glycolytic pathway, respectively. 2-DG, at non-toxic doses, had no effect on Th17 differentiation or IL-17 production but significantly reduced Treg proportions. In contrast, FX11 significantly reduced Th17 differentiation and IL-17 production without significant effect on Treg cell differentiation. These results suggest that lactate dehydrogenase activity is essential for the differentiation of Th17 cells but dispensable for Treg cell differentiation, identifying it as a potential target for the treatment of Th17-induced inflammation in disease. LDH’s relevance extends beyond autoimmune diseases. Elevated LDH activity is a hallmark of many cancers, where it fuels the Warburg effect, supports tumor growth, and promotes adaptation to hypoxia (37). In the tumor microenvironment, lactate accumulation enhances Treg function and suppresses effector T cell responses, contributing to immune evasion (38). Similarly, pathogens such *Staphylococcus aureus*, produce lactate that drives Treg expansion and IL-10 production, dampening host immunity (39). In autoimmunity, LDH is elevated in several types of inflammatory immune cells and is an important driver for promoting their pathogenic function (40, 41).

In summary, IL-6R is a key regulator of T cell homeostasis and Th17 cell differentiation, which is mediated at least in part through metabolic reprogramming. IL-6R signaling promotes both glycolysis and oxidative capacity in Th17 cells, and its effects are largely mediated through STAT3. Targeting the metabolic mediators of IL-6R signaling in T cells provides a promising strategy for modulating Th17-induced inflammation. Due to the reversible nature of targeting metabolic enzymes with small molecule inhibitors, targeting the metabolic enzymes induced by IL-6 can be used to fine tune inflammation associated with autoimmune disease or viral infection without causing deleterious effects on protective immunity.

## Data Availability

The original contributions presented in the study are included in the article/**Supplementary Material**. Further inquiries can be directed to the corresponding author.
