# Supplementary material for: Targeting IL-6 receptor mediated metabolic pathways to control Th17 cell differentiation and inflammatory responses
Source: Front Immunol. 2025 Aug 27;16:1568514. doi: 10.3389/fimmu.2025.1568514 (PMC12420212; doi:10.3389/fimmu.2025.1568514)
Supplement: Supplementary file 2 [file DataSheet1.pdf]

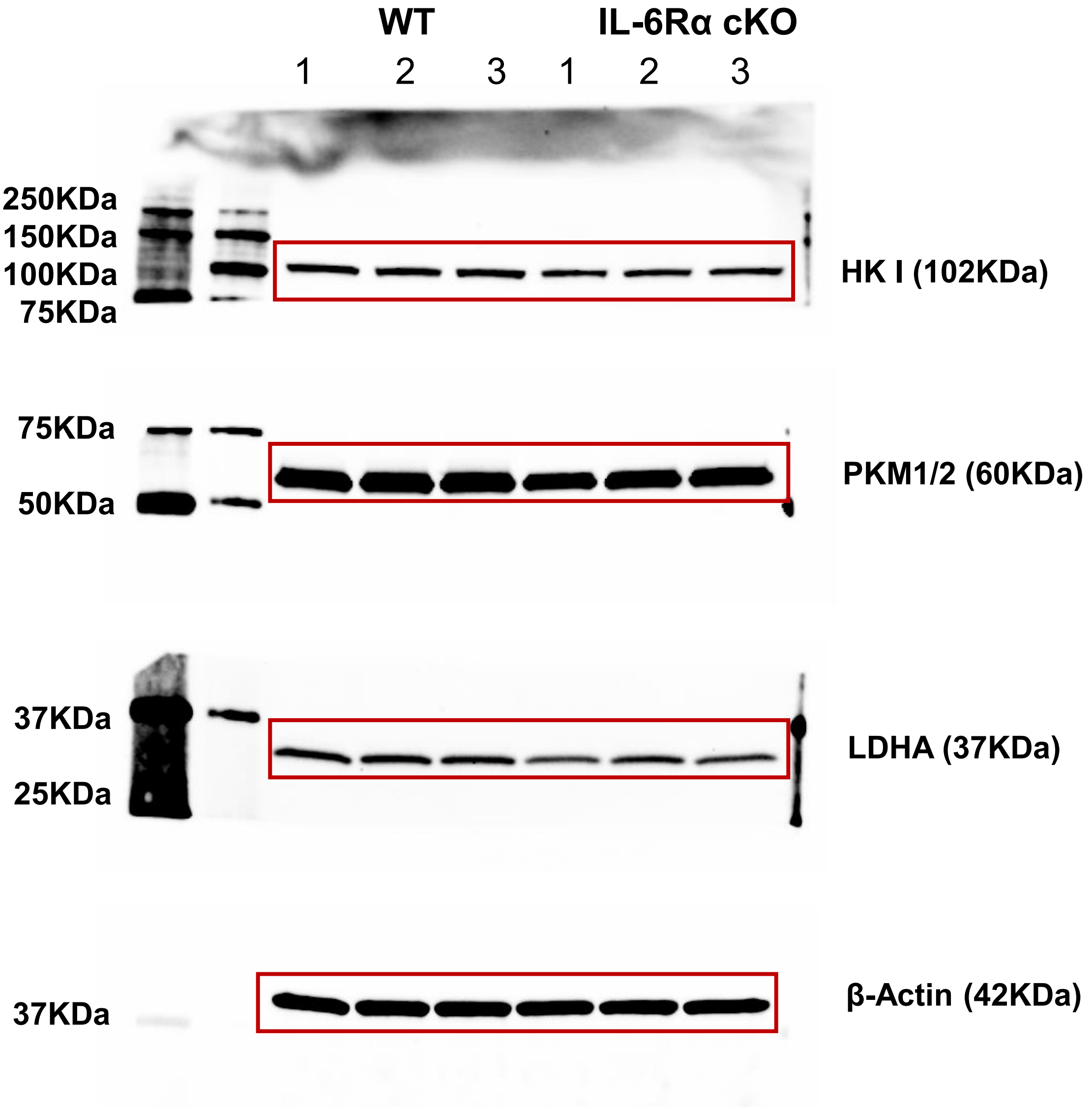

Original blots for figure 7 A

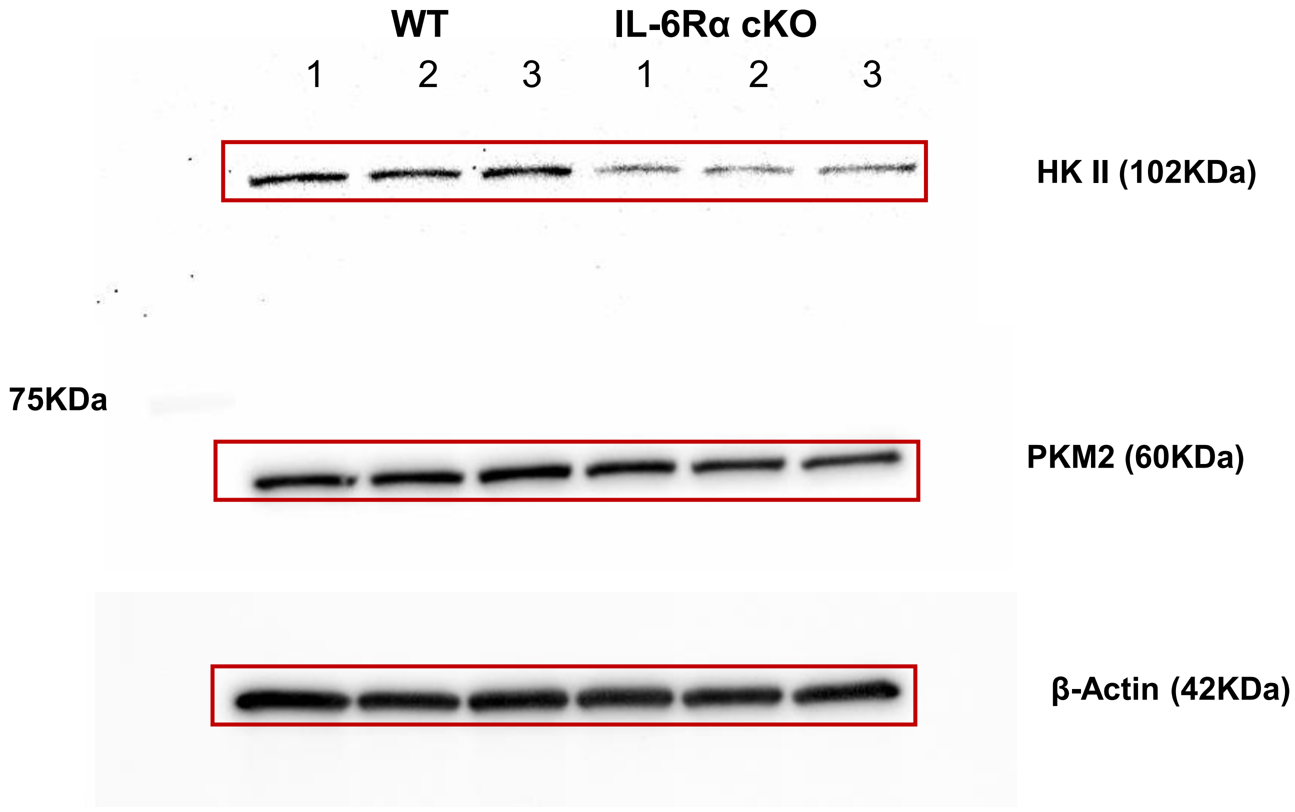

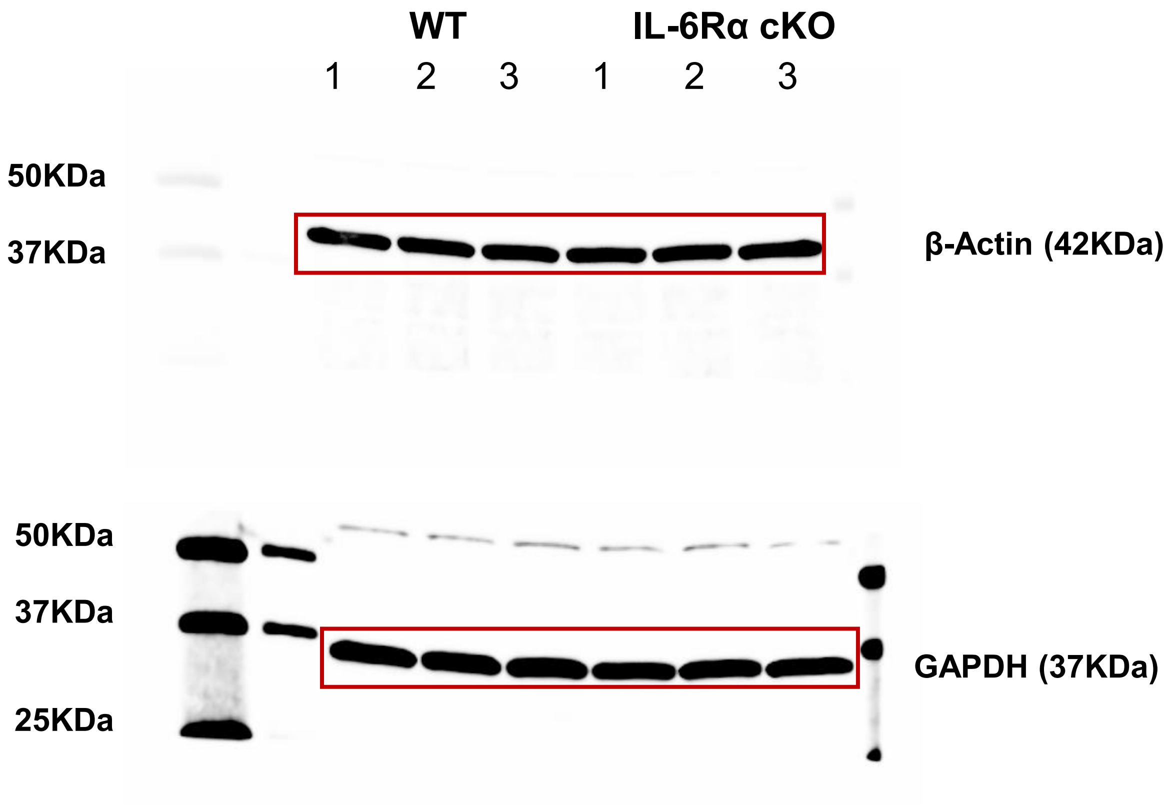

Original blots for figure 7 B

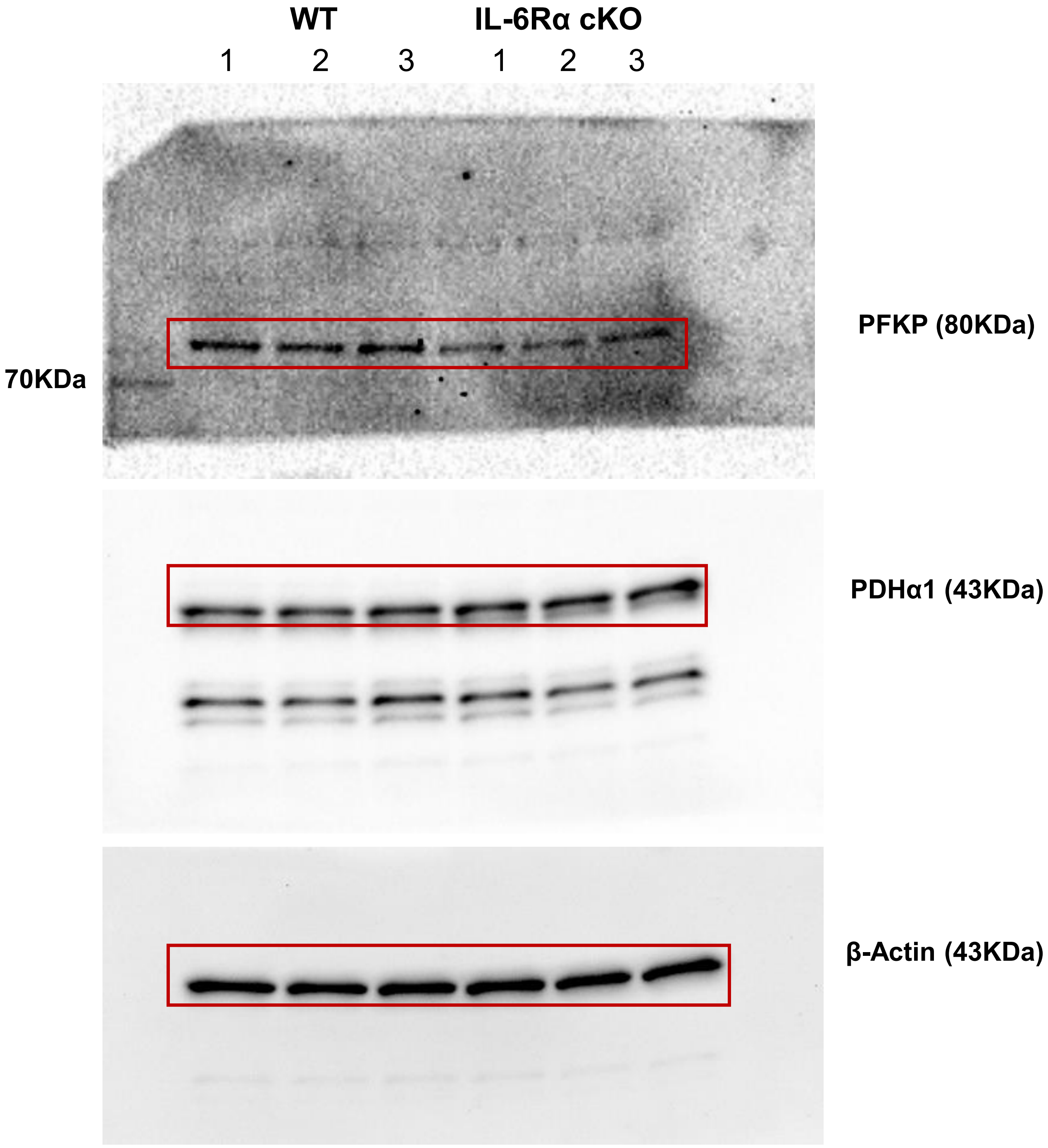

Original blots for figure 7 C

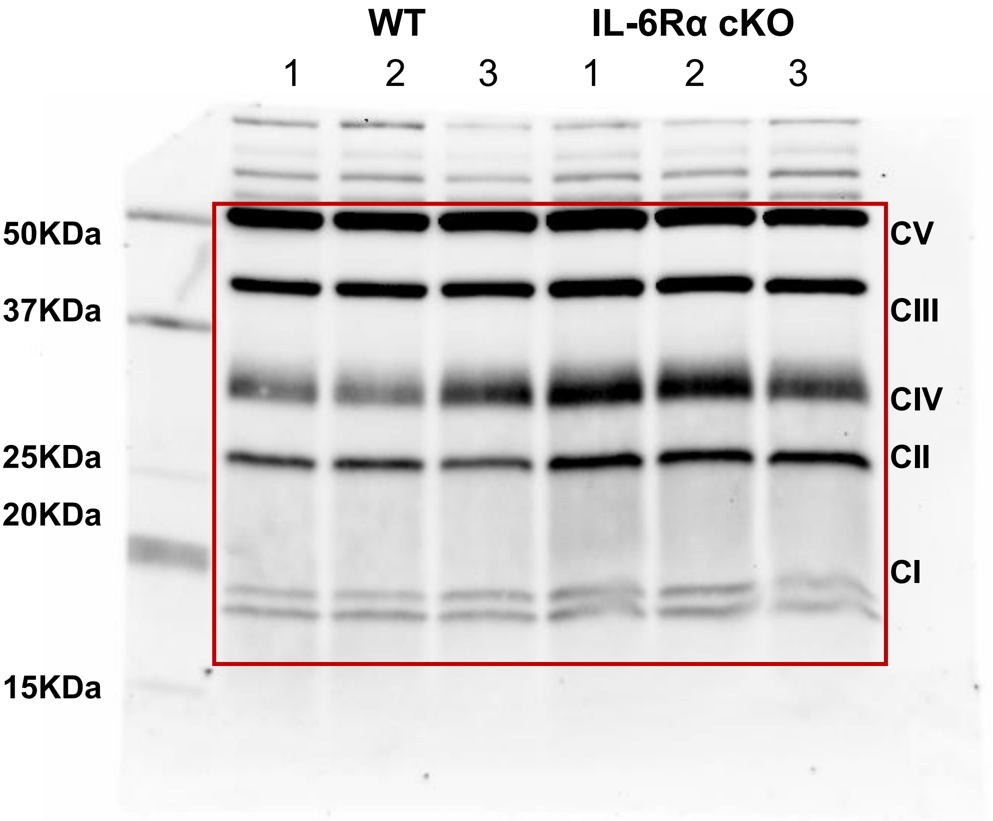

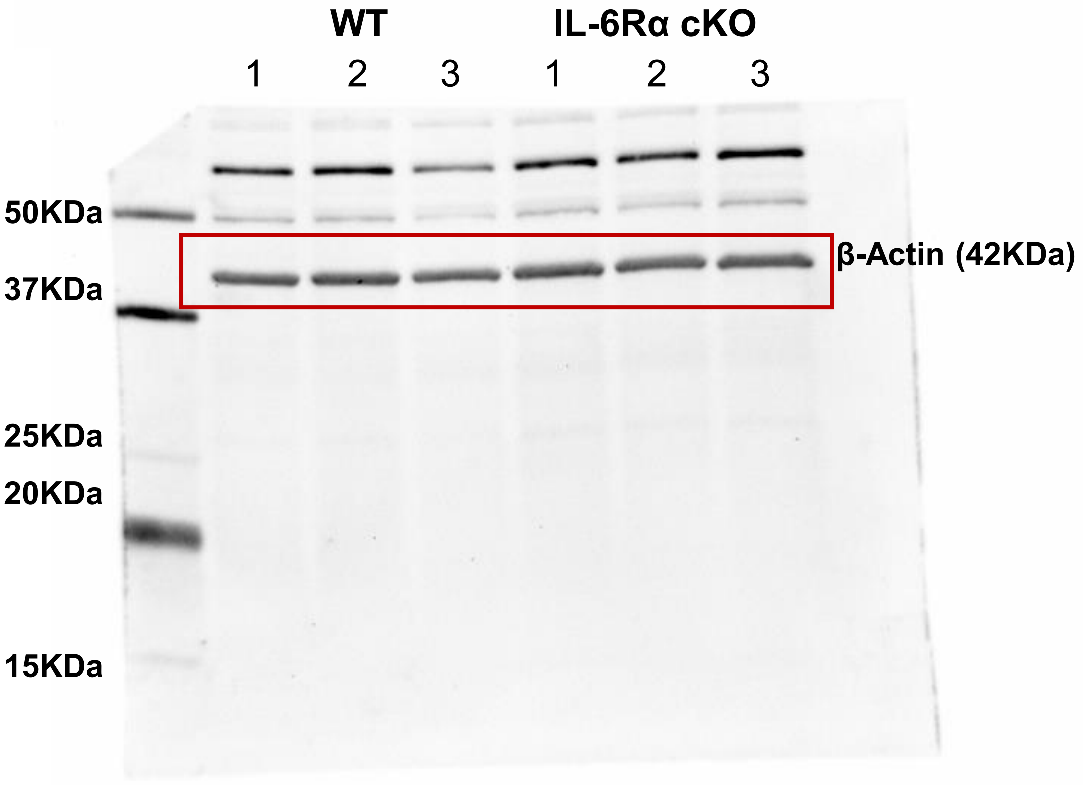

Original blots for figure 7 D
