# Supplementary material for: Targeting IL-6 receptor mediated metabolic pathways to control Th17 cell differentiation and inflammatory responses
Source: Front Immunol. 2025 Aug 27;16:1568514. doi: 10.3389/fimmu.2025.1568514 (PMC12420212; doi:10.3389/fimmu.2025.1568514)
Supplement: Supplementary file 3 [file DataSheet2.pdf]

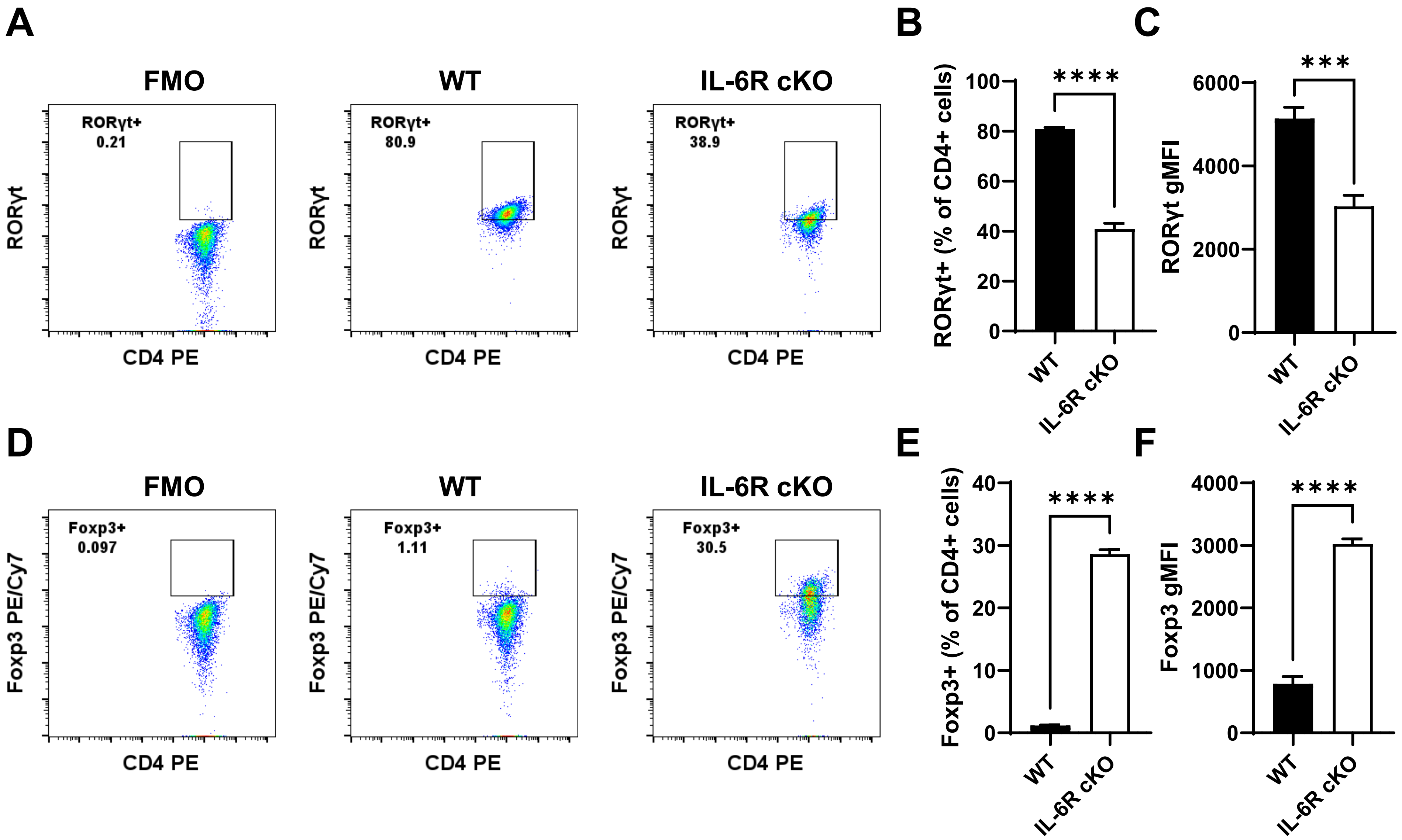

Supplementary Figure 1. Loss of IL-6 signal increases Treg differentiation under Th17 differentiation conditions. CD4 T cells were isolated from IL-6R T cell specific knockout and WT mice and differentiated under Th17 conditions for 72 hours and stained for RORγt and Foxp3 transcription factors and analyzed by flowcytometry. **(A)** Representative plot for intracellular staining of RORγt. **(B)** Proportion of CD4 T cells expressing RORγt. **(C)** Quantification of RORγt expression. **(D)** Representative plot for intracellular staining of Foxp3. **(E)** Proportion of CD4 T cells expressing Foxp3. **(F)** Quantification of Foxp3 expression. Results pooled from 2 independent experiments (n=6). Error bars represent ± SEM. T-test with Welch's correction or Mann Whitney test was used to compare groups depending on the normality of the distribution as judged by the Shapiro–Wilk test. *** $p < 0.001$, **** $p < 0.0001$.
